# Crosstalk between Venous Thromboembolism and Periodontal Diseases: A Bioinformatics Analysis

**DOI:** 10.1155/2021/1776567

**Published:** 2021-12-10

**Authors:** Zheng He, Qilong Jiang, Fuping Li, Mingxiang Chen

**Affiliations:** Department of Cardiovascular Surgery, The Third Affiliated Hospital of Chongqing Medical University, Shuanghu Branch Road No. 1, Yubei District, Chongqing 401120, China

## Abstract

**Background:**

This current study applied bioinformatics analysis to reveal the crosstalk between venous thromboembolism (VTE) and periodontitis, as well as the potential role of immune-related genes in this context.

**Methods:**

Expression data were downloaded from the GEO database. Blood samples from venous thromboembolism (VTE) were used (GSE19151), while for periodontal disease, we used gingival tissue samples (GSE10334, GSE16134, and GSE23586). After batch correction, we used “limma” packages of R language for differential expression analysis (*p* value < 0.05, ∣logFC | ≥0.5). We used Venn diagrams to extract the differentially expressed genes common to VTE and periodontitis as potential crosstalk genes and applied functional enrichment analysis (GO biological process and KEGG pathway). The protein-protein interaction (PPI) network of crosstalk genes was constructed by Cytoscape software. The immune-related genes were downloaded from the literature. The Wilcoxon test was used to test the scores of immune infiltrating cells. The crosstalk genes were further screened by LASSO Logistic Regression.

**Results:**

For periodontitis, 427 case and 136 control samples, and for VTE, 70 case and 63 control samples were included. The obtained PPI network had 1879 nodes and 2257 edges. Moreover, 782 immune genes and 28 cell types were included in the analysis. Over 90% of immune cells had different expressions in VTE and periodontitis. We obtained 12 significant pathways corresponding to crosstalk genes. CD3D, CSF3R, and CXCR4 acted as an immune gene and a crosstalk gene. We obtained a total of 12 shared biomarker crosstalk genes. Among those 12 biomarker crosstalk genes, 4 were immune genes (LGALS1, LSP1, SAMSN1, and WIPF1).

**Conclusion:**

Four biomarker crosstalk genes between periodontitis and VTE were also immune genes, i.e., LGALS1, LSP1, SAMSN1, and WIPF1. The findings of the current study need further validation and are a basis for development of biomarkers.

## 1. Introduction

Venous thromboembolism (VTE) summarizes deep vein thrombosis and pulmonary embolism, showing an annual incidence of 1-2/1000 individuals, making VTE the third most common cardiovascular disease in the world [1, 2]. Due to the increasing life expectancy and thus age of the population, a growing disease burden of VTE can be expected [3]. Risk management and respective therapeutic as well as preventive control, especially by appropriate anticoagulation strategy, appear of high importance in respective patients [4]. Thereby, the appropriate preventive care is still controversially discussed [4]. The risk factors for VTE are comparable as for atherosclerosis, including obesity, smoking, and diabetes alongside with hypertension and hypercholesteremia as well as hyperlipidemia [5].

Taking the recent literature into account, periodontal diseases seem to be a further risk factor for VTE; it has been found that patients with periodontitis had an increased relative risk to develop VTE, with an RR ranging between 1.46 and 1.91 (overall 1.61) [6–8]. Periodontitis is an inflammatory disease of the gums and tooth surrounding bone, resulting in tooth loss at the end-stage [9]. In these inflammatory diseases, pathogenic bacteria were reported to play a crucial role, including different gram-negative anaerobes with a high virulence and thus pathogenic potential [9]. Caused by the bacteremia of these pathogens related to periodontitis as well as a systemic inflammatory reaction, periodontal diseases are closely related to cardiovascular diseases and atherosclerosis [10, 11]. Moreover, periodontitis is associated with main risk factors, including smoking, diabetes, obesity, and low compliance [9], which are quite similar as for VTE [5].

Although a relationship between periodontitis and vascular diseases, alongside with shared risk factors, is known, the underlying mechanisms are not fully understood, yet. However, a deeper understanding, especially developing respective diagnostic and/or therapeutic biomarkers, would be preferable for future management of these diseases. Therefore, this current study is aimed at revealing the crosstalk between VTE and periodontitis, as well as the potential role of immune-related genes in this context. The underlying purpose was to investigate whether there exist potentially shared biomarker genes for the two diseases. It was hypothesized that there would be potential crosstalk biomarker genes, which are shared between periodontitis and VTE.

## 2. Materials and Methods

### 2.1. Datasets

Expression data of VTE and periodontal disease (PD) were downloaded from the GEO database (https://www.ncbi.nlm.nih.gov/geo/). Blood samples from VTE were used, wherefore we obtained GSE19151. For PD, we used gingival tissue samples. To facilitate subsequent analysis, we screened datasets with the same platform and finally obtained datasets GSE10334, GSE16134, and GSE23586 (Table 1).

### 2.2. Data Preprocessing and Differential Expression Analysis

Firstly, for chip data, we converted probe ID into gene symbol according to their platform information. For the data of multiple probes corresponding to the same gene, the mean value of the sample was used as the gene expression value of the sample.

Since for PD three datasets were available, that information was combined; to reduce the differences in the combination of batches of samples, we used the ComBat method in R's SVA package to conduct batch correction of the combined data. The resulting PD-related dataset contained 427 case and 136 control samples. Subsequently, we performed PCA analysis on the expression values of the samples before and after correction.

We used “limma” packages of R language for differential expression analysis of case and control samples of VTE and PD, respectively. Differential expression was defined as follows: *p* value < 0.05, ∣logFC | ≥0.5 for differentially expressed genes (DEG), including logFC ≥ 0.5 for upregulated genes and logFC ≤ 0.5 for downregulated genes.

### 2.3. Crosstalk Gene Analysis

We used Venn diagrams to extract the differentially expressed genes common to VTE and PD as potential crosstalk genes. To further analyze the functions of crosstalk genes, clusterProfiler of R language was used for functional enrichment analysis (GO biological process and KEGG pathway), and the functions with *p* value < 0.05 were screened as significant functions.

### 2.4. Crosstalk Gene-Related PPI Network

We downloaded experimental protein-protein interaction relationship (PPI) pairs from the databases listed in Table 2.

Then, the PPI relationship pairs of crosstalk genes were extracted, and the PPI network of crosstalk genes was constructed by Cytoscape software. At the same time, we used Network Analyzer to analyze the average shortest path length, betweenness, and total degree of network.

### 2.5. Immunoinfiltration Analysis of Immune Genes for VTE and PD

The immune-related genes were downloaded from the literature (PMID: 28052254). The geneset contained 782 genes and 28 cell types, and the immune types included both adaptive and innate. There were 15 adaptive immune cells and 13 innate immune cells. Firstly, respective genes appearing together in PD and VTE were screened; then, the expression values of these immune genes shared by PD and VTE in the case samples were extracted. Combined with the cells corresponding to the genes, the ssGSEA algorithm was used to analyze the infiltration of immune cells. At the same time, the Wilcoxon test was used to test the scores of immune infiltrating cells in case samples of VTE and PD in two datasets to see whether there was a significant relationship between the same immune cells in the two diseases (*p* < 0.05).

### 2.6. Crosstalk Genes and TF Network

We used TRRUST (https://www.grnpedia.org/trrust/), cGRNB (https://www.scbit.org/cgrnb), HTRIdb (http://www.lbbc.ibb.unesp.br/htri/), ORTI (http://orti.sydney.edu.au/about.html), and TRANSFAC (http://gene-regulation.com/pub/databases.html) to download transcription factors and target gene relations. We extracted the TF corresponding to crosstalk genes and established the TF-target network using Cytoscape software. Afterwards, the topological properties of TF-target network were analyzed using Cytoscape plug-in Network Analyzer.

### 2.7. Pathway Relationships between Crosstalk Genes and Immune Genes

We obtained significant pathways corresponding to crosstalk genes through functional enrichment analysis. Then, we obtained all genes under these pathways from the KEGG database. At first, we marked the types of these genes and then built a pathway gene network based on these gene attributes, using the respective pathway as a bridge to discover the relationship between crosstalk genes and immune genes.

### 2.8. Crosstalk Genes Screened by LASSO Regression Analysis

In order to screen out the most relevant crosstalk genes, we used the expression values of those crosstalk genes in VTE and PD as characteristic values and applied the “glmnet” package of R project for analysis. The crosstalk genes were further screened by LASSO Logistic Regression. Then, the crosstalk genes of PD and VTE obtained by LASSO regression analysis were set as the intersection, and the shared crosstalk genes were labeled as potential biomarkers. Then, the expression values of the potential biomarker crosstalk genes in all VTE and PD samples were extracted and the Wilcoxon test was performed, indicating that they were significant between the disease samples and control samples. Finally, ROC analysis was performed on the potential biomarker crosstalk genes to predict their predictive efficiency for disease.

## 3. Results

### 3.1. Differential Expression Analysis

**Figure 1:**
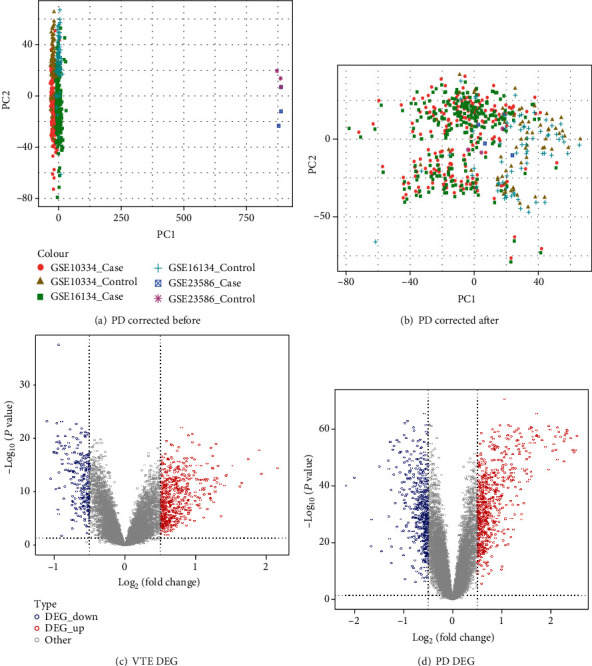
Data preprocessing and differential expression analysis: (a) PCA results before correction of gene expression profile in PD dataset; (b) PCA results after gene expression profile correction in PD dataset; (c) PCA results after gene expression profile correction in PD dataset; (d) volcano map of PD differentially expressed genes.

We combined the expression profiles obtained from PD and performed batch correction. Then, we conducted PCA analysis on the expression values of the samples before and after correction and found that there were differences in the samples before correction, while differences between the samples after correction had decreased (Figures 1(a) and 1(b)).

Subsequently, we performed the differential expression analysis for VTE and corrected data of VTE using R language limma. The volcanic map shows the respective gene distribution (Figures 1(c) and 1(d)). The number of differentially expressed genes obtained is shown in Table 3.

### 3.2. Crosstalk Genes

**Figure 2:**
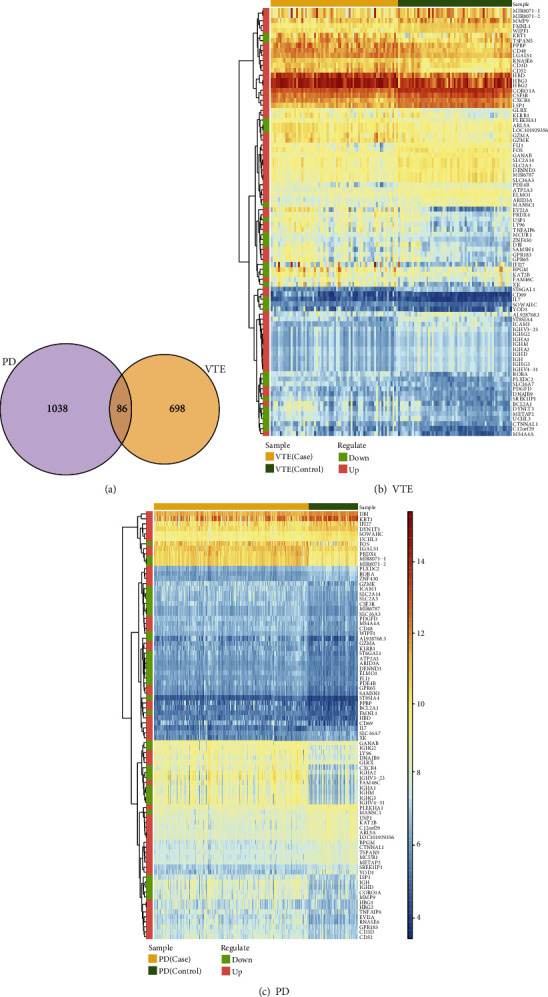
(a) Venn diagram of differentially expressed genes of VTE and PD. (b, c) Heatmaps of crosstalk genes in VTE and PD.

We used Venn diagrams to extract the differentially expressed genes common to VTE and PD as potential crosstalk genes (Figure 2(a)). In order to observe the types of crosstalk genes and the changes of expression values of these genes in different types of samples, we used the heat map package of R language to draw heat maps (Figures 2(b) and 2(c)).

**Figure 3:**
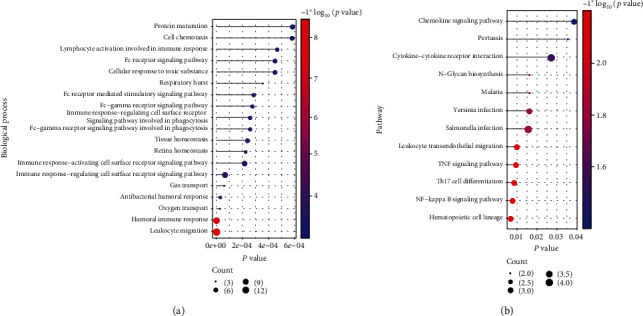
The functions regulated by crosstalk genes: (a) the biological process of crosstalk gene enrichment; (b) all significant KEGG pathways regulated by crosstalk genes.

To further analyze the functions of crosstalk genes, clusterProfiler of R language was used for functional enrichment analysis (GO biological process and KEGG pathway), and the functions with *p* value < 0.05 were screened as significant functions. The results are displayed in Figures 3(a) and 3(b).

### 3.3. Crosstalk Gene-Related PPI Network

**Figure 4:**
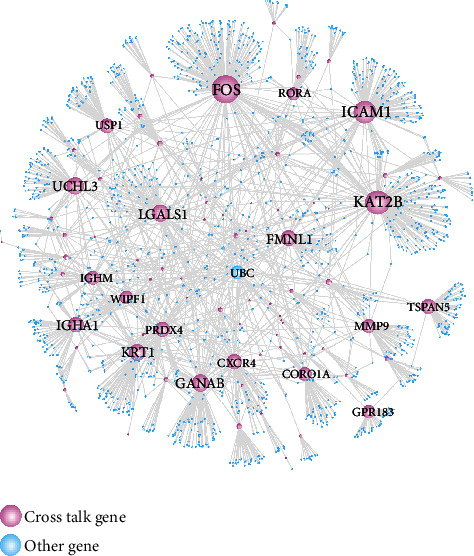
Crosstalk gene-related PPI network.

We extracted the PPI relationship pairs of crosstalk genes from the public database and constructed the PPI network (Figure 4) using Cytoscape software. The obtained network had 1879 nodes and 2257 edges.

At the same time, we used Cytoscape to conduct topological property analysis on the network. In the analysis results, we screened the top 20 genes (Table 4) as important hub node genes. It can be seen from the results that crosstalk genes FOS, T2B, and CAM1 appear to play important roles in the whole biological network.

### 3.4. Immune Infiltration Analysis of Immune Genes

**Figure 5:**
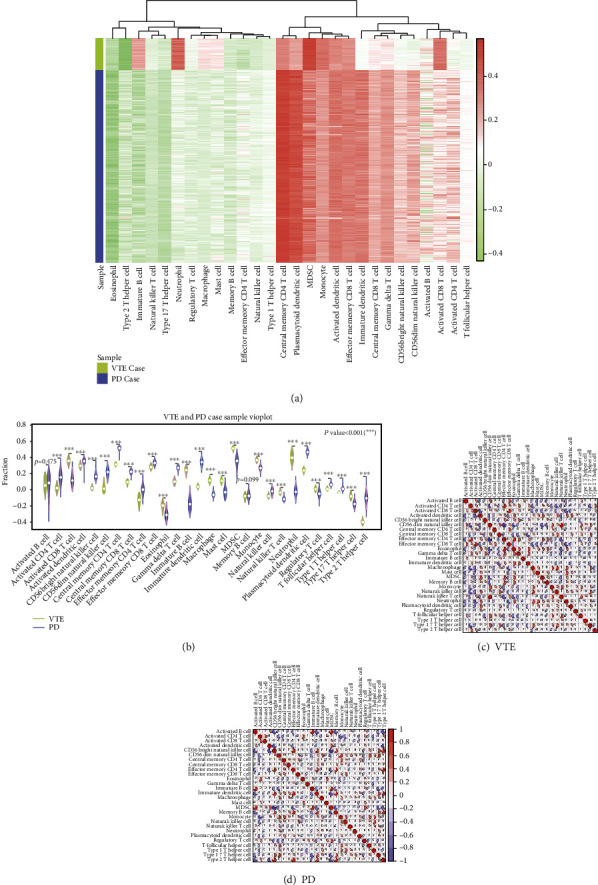
Expression of immune genes in VTE and PD: (a) the immune infiltration level of immune genes in VTE and PD datasets; (b) differences in immune cell expression between VTE and PD; (c, d) correlation of immune cells in VTE and PD.

In order to analyze the role of immune genes in VTE and PD, we downloaded immune-related genes from reference (PMID: 28052254). After the results of immune cell scores were obtained, we used the R heat map to display the scores of immune infiltrating cells in VTE and PD datasets (Figure 5(a)) to check the expression levels of immune cells. The results showed that central memory CD4 T cells, plasmacytoid dendritic cells, activated dendritic cells, and effector memory CD8 T cells were highly expressed in VTE and PD. Immature B cells and neutrophils were highly expressed in the VTE and lowly expressed in the PD.

We used the “vioplot” package of R to draw a violin diagram to show the score distribution of each immune cell in the two diseases. Meanwhile, the Wilcoxon test was used to test the scores of immune infiltrating cells in case samples of VTE and PD in the two datasets, and the significant relationships between the same immune cells in the two diseases (*p* < 0.05) were screened (Figure 5(b)). It can be seen that over 90% of immune cells have different expressions in VTE and PD.

In order to check the relationship between immune cells in VTE and PD, we analyzed the correlation of immune cells and used R's “corrplot” package to display the analysis results, so as to check whether the correlation trend among immune cells in different diseases is consistent (Figures 5(c) and 5(d)). In VTE, CD56dim natural killer cells were highly positively correlated with natural killer T cells (COR = 0.8020); activated CD8 T cells were positively correlated with effector memory CD8 T cells (COR = 0.7058). In PD, activated B cells were highly positively correlated with MDSC (COR = 0.7742), CD56bright natural killer cells were highly positively correlated with Type 2 T Helper cells (COR = 0.7697), and effector memory CD4 T cells were highly positively correlated with Type 2 T helper cells (COR = 0.7654).

### 3.5. TF-Regulated Crosstalk Genes and Immune Gene Analysis

**Figure 6:**
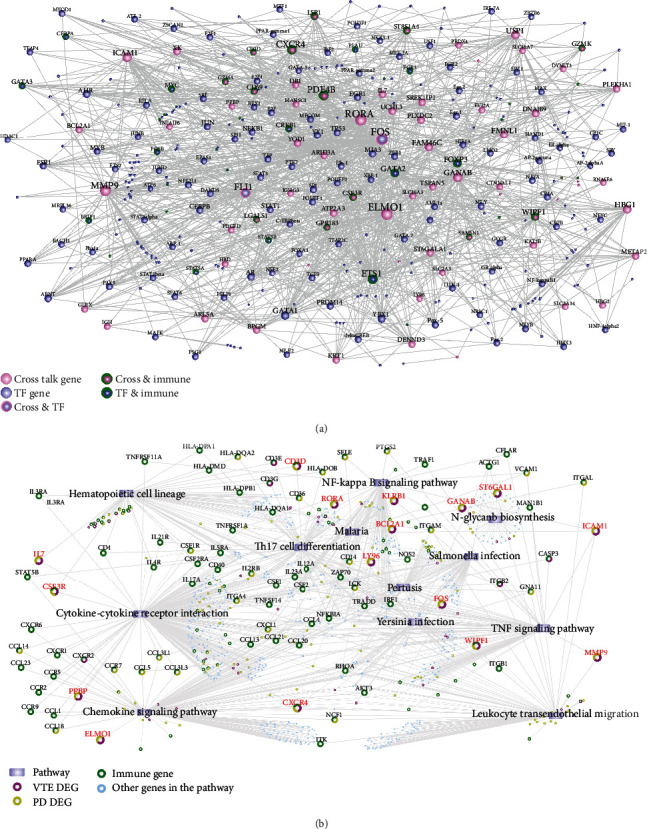
A network of biological functions related to crosstalk genes: (a) TF-crosstalk gene network; (b) pathway crosstalk gene/immune gene network.

After downloading the relationship between the transcription factors and the target genes from the TF-related database, we extracted the TF corresponding to crosstalk genes. The TF-target network (Figure 6(a)) was established by Cytoscape software, and the topological properties of the TF-target network were analyzed. This network is composed of 374 nodes and 1279 edges. We also mapped 685 immune genes into TF-crosstalk gene network and showed the important nodes to illustrate the relationships among TF, crosstalk genes, and immune genes according to the results of topology analysis.

From the network, we can obtain that FOS and FLI1 are both crosstalk genes and TF and regulate other genes to affect biological functions. In addition, genes such as ETS1, FOXP3, and GATA2 are both immune genes and TF and affect immune function by regulating other genes.

### 3.6. Pathway Relationships between Crosstalk Genes and Immune Genes

We obtained 12 significant pathways corresponding to crosstalk genes (Figure 3(b)). Now, we obtained all genes under these pathways from KEGG database, and these genesets may contain any combination of PD DEG, VTE DEG, immune genes, and other genes in the pathway. We first identified the type of genes to which they belonged. Then, a pathway-gene network (Figure 6(b)) was established based on these gene attributes, and pathways were used as a bridge to discover the relationship between crosstalk and immune genes. As shown in the figure, CD3D was the crosstalk gene of VTE and PD, and at the same time, immune gene, regulating pathways, hematopoietic cell lineage, and Th17 cell differentiation. In addition, CSF3R is both a crosstalk gene and an immune gene, regulating cytokine-cytokine receptor interaction and hematopoietic cell lineage. CXCR4 acts as an immune gene and crosstalk gene, regulating the pathways of cytokine-cytokine receptor interaction, leukocyte transendothelial migration, and chemokine signaling pathway. Genes in the pathway, crosstalk genes, and immune genes jointly affect the occurrence of VTE and PD, providing the possibility of crosstalk between them.

### 3.7. LASSO Logistic Regression of Crosstalk Genes

**Figure 7:**
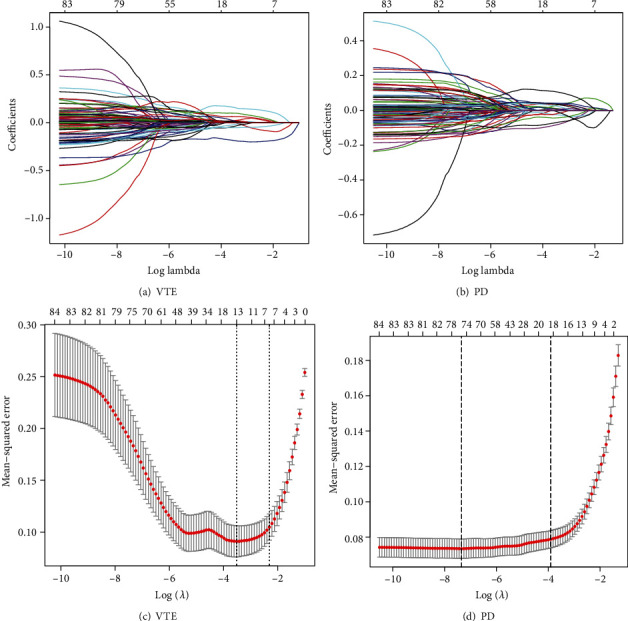
(a, b) Change curves of characteristic gene for VTE and PD. The abscissa (below) is the logarithm of the lambdas, the ordinate is the variable coefficient, and the abscissa (above) is the remaining number of variable genes whose variable coefficient is not 0 under the log value of the current lambda. It can be seen that with the increase of the abscissa lambdas value, the coefficient of variables decreases continuously, and some coefficients of variables become 0, while the later characteristic genes approaching 0 are more important in the dataset. (c, d) The results of cross-checking the lambda result. There are two dashed lines in the figure: one is lambda.min with the minimum mean square error, and the other is lambda.1se with the standard error from the minimum mean square error.

We obtained 86 crosstalk genes, and there were many immune genes under these crosstalk genes. LASSO Logistic Regression was used to further screen crosstalk genes based on the glmnet package of R (Figures 7(a)–7(d)).

Through screening, we obtained a total of 13 crosstalk genes in VTE and 75 crosstalk genes in PD, and they shared 12 crosstalk genes. These 12 potential biomarker crosstalk genes were obtained by LASSO Logistic Regression. Among the 12 potential biomarker crosstalk genes, 4 were immune genes (LGALS1, LSP1, SAMSN1, and WIPF1).

**Figure 8:**
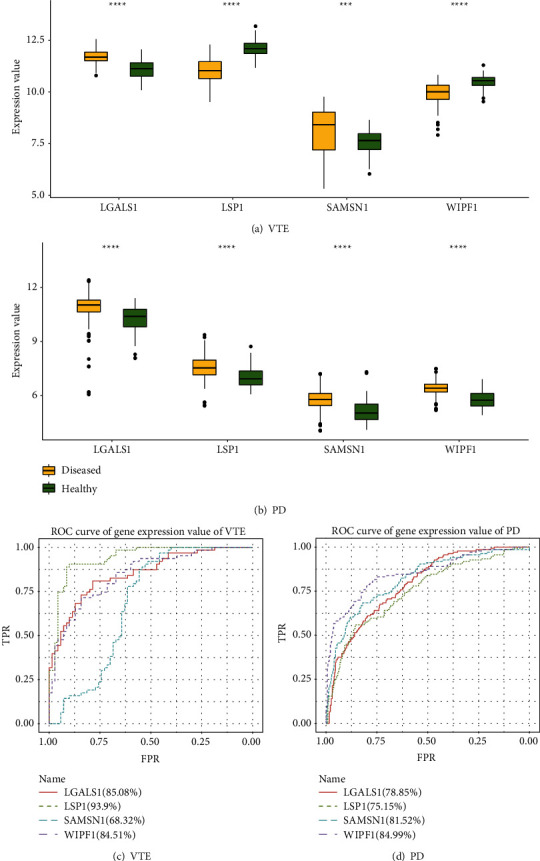
(a) Expression levels of LGALS1, LSP1, SAMSN1, and WIPF1. (b) ROC analysis results of LGALS1, LSP1, SAMSN1, and WIPF1.

Firstly, the expression values of these 4 genes in all samples of VTE and PD were displayed in boxplots and the Wilcoxon test (Figures 8(a) and 8(b)) was conducted. It was found that they had a high significance between disease and normal samples. Then, we performed ROC analysis on 4 of them (Figures 8(c) and 8(d)) and found that SAMSN1 was slightly lower in VTE. The AUC of LGALS1, LSP1, and WIPF1 in VTE and PD were all greater than 75%. The results showed that these expression values were reliable for gene analysis.

**Figure 9:**
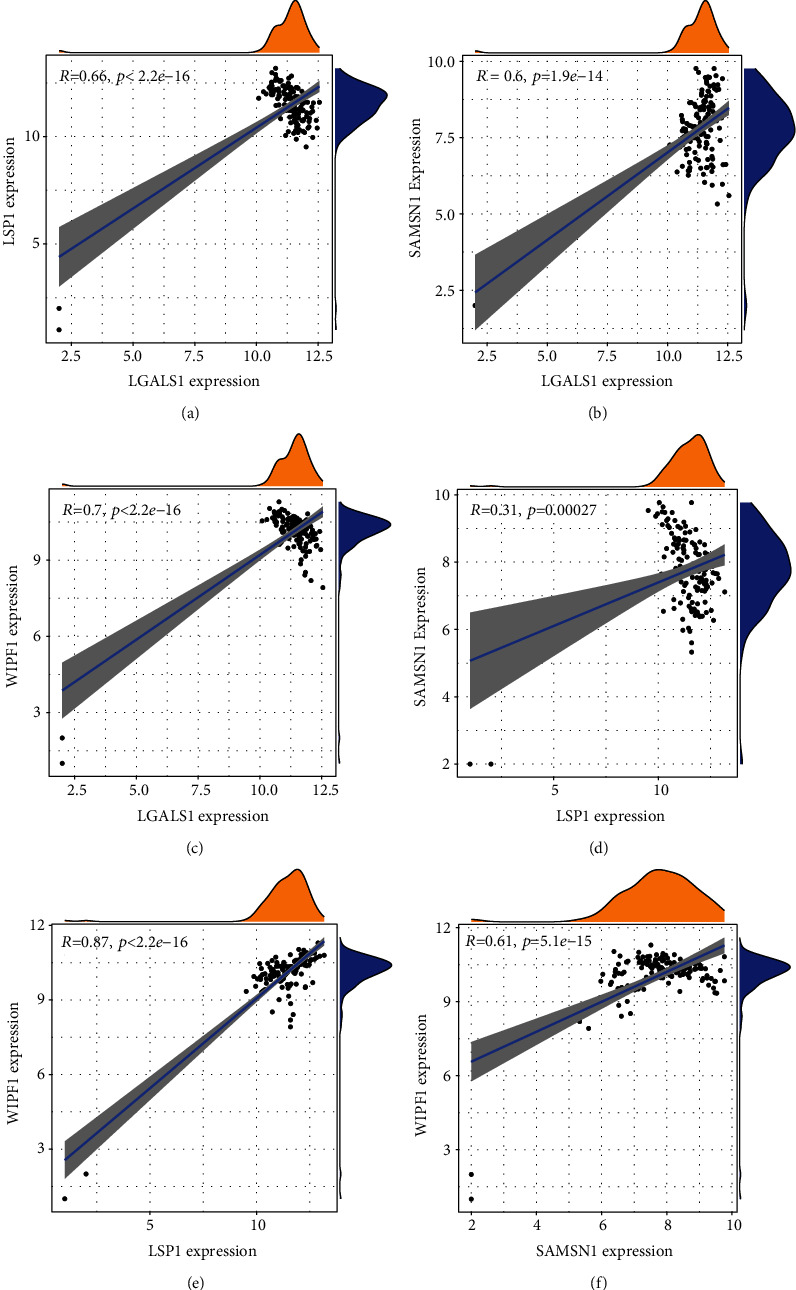
Correlation among LGALS1, LSP1, SAMSN1, and WIPF1 in VTE.

**Figure 10:**
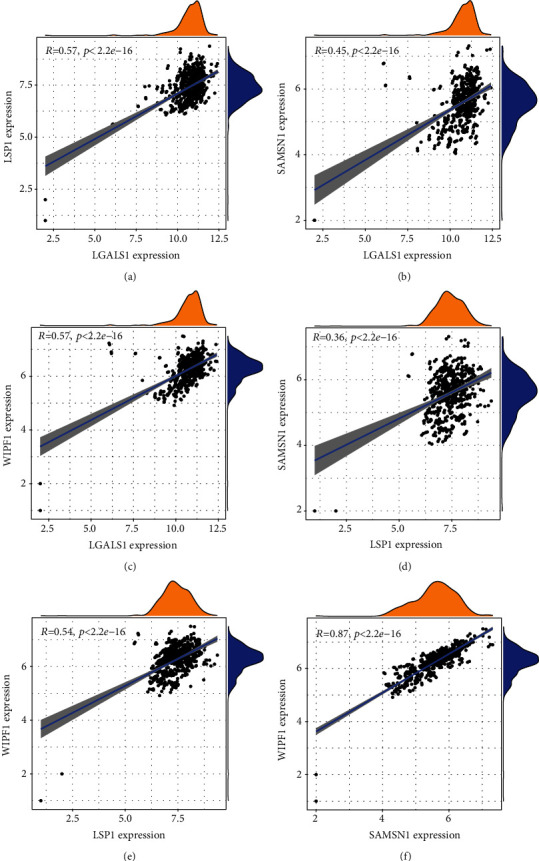
Correlation among LGALS1, LSP1, SAMSN1, and WIPF1 in PD.

Finally, we checked the correlation between any pair of four genes and found that there was also a close relationship between them in terms of expression level. Results showed a high correlation between LSP1 and WIPF1 in VTE (COR = 0.8652) (Figures 9(a)–9(f)). SAMSN1 and WIPF1 were highly correlated in PD (COR = 0.8689) (Figures 10(a)–10(f)).

## 4. Discussion

Main results: based on the variety of results and their complexity, this discussion will focus on the main findings and their potential clinical relevance. Three crosstalk genes, CD3D, CSF3R, and CXCR4, were also immune genes and involved in different pathways. Among the 12 potential biomarker crosstalk genes, 4 were immune genes, i.e., LGALS1, LSP1, SAMSN1, and WIPF1, showing a high correlation between LSP1 and WIPF1 in VTE and between SAMSN1 and WIPF1 in PD. Accordingly, the previously formed hypothesis was confirmed.

At first, it appears most reasonable to focus on the revealed potential biomarker crosstalk genes. LGALS1, i.e., lectin, galactoside-binding, soluble, 1, is an important molecule in different signaling pathways and immune response [12]. The important role of galectin-1 has been repeatedly discussed in context of periodontal diseases; thereby, it was reported to be mainly involved in lipopolysaccharide-related reaction of periodontal ligament cells [13]. In this context, proteins originating from the galectin superfamily were found to be involved in the reaction against periodontal pathogenic bacterial biofilm [14]. It was reported that galectin-1 would be able to enhance the epithelial invasion of the oral epithelial cells by *Porphyromonas gingivalis*, a major periodontal pathogen [15]. It has been shown that this *Porphyromonas gingivalis* activates platelet Cdc42 and promotes platelet spreading and thrombosis [16]. This underlines the important role of periodontal bacteria in cardiovascular diseases and thrombosis development [9]. Although there are no results regarding LGALS1 and VTE, this potential biomarker crosstalk gene could be a hint for a bacterial interlink, i.e., the epithelial invasion of periodontal pathogens resulting in inflammation and thromboembolism as a systemic effect.

The lymphocyte-specific protein 1 (LSP1) has an important role in neutrophil motility, fibrinogen matrix protein adhesion, and transendothelial migration [17]. No data for periodontitis as well as VTE are available for this, making any conclusions on its relevance difficult. Although this remains speculative, the functions of LSP1 appear to support its role in the relationship between periodontitis and VTE; similarly, as for atherosclerotic diseases, fibrinogen matrix protein adhesion might be related to oxidative stress and inflammatory dysfunction in context of periodontal inflammation, while the transendothelial migration supports the potential role of invading periodontal pathogens [18]. SAMSN1 is a cytoplasmic adaptor protein, predominantly expressed in the hematopoietic compartment, which is associated with adaptive immune response, as well as B-cell activation and differentiation [19–21]. Similar as for LSP1, no studies reported the role of SAMSN1 in periodontitis or VTE, yet. B cells are crucial in controlling the chronic inflammatory processes during periodontal diseases [22]. Therefore, the relation between SAMSN1 and periodontitis appears plausible. A differential expression of the platelet gene SAMSN1 has been reported to be related to myocardial infarction [23], and SMSN1 was also associated with coronary atherosclerosis [24]. Although the pathogenesis of VTE and atherosclerosis is basically different, it argues for the periocardiovascular relationship. Lastly, the Wiskott-Aldrich syndrome protein-interacting protein family member 1 (WIPF1) is a protein primarily related to invasion and metastasis of different malignancies [25]. No studies reported on a potential role of WIPF1 in periodontitis or VTE. However, WIPF1 was differentially expressed in smokers with lung carcinoma [26] and might therefore be related to smoking-induced changes, which are a shared risk factor for periodontitis and VTE [5, 9]. However, this remains a speculative hypothesis, needing further validation.

Three genes were both crosstalk and immune genes. Of these, colony-stimulating factor receptor 3 (CSFR3) was found to be involved in periodontal diseases; thereby, the colony-stimulating factor 2, which is originating from the same superfamily, contributed to the regulation of inflammatory response during periodontal homeostasis [27]. Moreover, CSFR1 was found to be related to diabetic periodontitis [28], potentially supporting the upper mentioned relevance of shared risk factors, because diabetes increases the risk for both periodontitis and VTE. On the other hand, the colony-stimulating factor was found to be relevant in context of vascular smooth muscle degeneration in context of cerebral thrombosis [29]. Thus, its relevance in the interplay between periodontitis and VTE appears conceivable. Additionally, CXC chemokine receptor 4 (CXCR4) was found to be related to periodontal diseases, especially in context of periodontal pathogens as *Porphyromonas gingivalis* [30, 31]. Furthermore, CXCR4 has also a regulatory role at vascular and tissue inflammation, immune defense, and repair in context of platelets [32]. This supports the interlink between periodontitis and VTE from the perspective of inflammation as well as bacterial invasion.

Altogether, the current bioinformatics study revealed several potential crosstalk biomarkers for the interrelation between periodontitis and VTE. Thereby, three mechanisms of interaction can be supported: (i) an influence of invading periodontal pathogens on the vascular system, (ii) an influence of the periodontitis-associated inflammation on platelet function and thrombosis risk, and (iii) an influence of shared risk factors like smoking and diabetes. These statements are just hypothetic, needing further validation. Moreover, the detailed mechanisms of action need to be further clarified and cannot be explained, yet. It is also unclear, which hypothesis would be the most relevant one in the interplay between periodontitis and VTE. Probably, a complex interplay between all these mechanisms would be responsible for the overlap between those two diseases. This discussion was focused on the main findings, i.e., the potential biomarker crosstalk genes. There were many findings in the current study, including biological pathways and processes, which are somewhat informative at the moment. Future studies will need to show the clinical significance of these results in the current analysis. Therefore, a detailed discussion was omitted, especially to not exceed the limits of a research article.

Strengths and limitations: this comprehensive and complex bioinformatics analysis addressed a recent and clinically interesting topic. However, several limitations need to be mentioned. The major point is the missing validation of the findings. While for periodontitis, 427 case and 136 control samples were included, for VTE, 70 case and 63 control samples were included. This imbalance in sample size limits the analysis and might indicate a shift in detected genes; Table 3 shows that there were more genes found for periodontitis, which might be related to this imbalance. This needs to be considered in the interpretation of the findings. Additionally, different sample types were analyzed (periodontitis: tissue; VTE: blood). Thereby, the direction of action remains unclear; it would be speculative to indicate whether the deregulation in periodontal tissue would enter the blood stream causing VTE or vice versa. This cannot be clarified based on the current analysis.

This analysis allows the identification of potential biomarkers and crosstalk genes based on available datasets; these findings rely on different patient cohorts, and the achieved relationships are not validated within the same individual. Thus, these findings would need experimental validation to allow robust conclusions. Therefore, all conclusions are just speculative; however, the findings can provide a theoretical framework and basis for future research. Additionally, all findings rely on the transcriptomic level. No information on the included patients was included. These would include age, gender, comorbidities, or smoking habits, as well as extent and severity of periodontitis. Thus, the generalizability of the findings is unclear. Lastly, information on patient-specific parameters is lacking and was not considered in this analysis. Altogether, future studies should examine the relationship between VTE and periodontitis based on the potential biomarker crosstalk genes in well-designed clinical studies. Thereby, patient- and disease-related factors need to be considered to allow generalizable results.

## 5. Conclusion

Four potential biomarker crosstalk genes were also immune genes, i.e., LGALS1, LSP1, SAMSN1, and WIPF1 between periodontitis and VTE. The findings of the current study need further validation and are a basis for development of biomarkers to gain insight into the interplay between periodontitis and VTE.

## Figures and Tables

**Table 1:** The datasets used for analysis.

Disease	Series	Platforms	Case	Control	Total
VTE	GSE19151	GPL571	70	63	133
PD	GSE10334	GPL570	183	64	247
	GSE16134	GPL570	241	69	310
	GSE23586	GPL570	3	3	6

**Table 2:** Protein-protein interaction databases.

Database	Link
HPRD	http://www.hprd.org/index_html
BIOGRID	http://thebiogrid.org/
MINT	https://mint.bio.uniroma2.it/
DIP	http://dip.doe-mbi.ucla.edu/dip/Main.cgi
Mentha	http://mentha.uniroma2.it/index.php
PINA	https://omics.bjcancer.org/pina/
InnateDB	http://www.innatedb.com/
INstruct	http://instruct.yulab.org/index.html

**Table 3:** The number of differentially expressed genes for VTE and PD.

Disease	Up	Down	Total
VTE	548	236	784
PD	663	461	1124

**Table 4:** Topological properties of top20 genes.

Gene	Label	Degree	Average shortest path length	Betweenness centrality	Closeness centrality	Topological coefficient
FOS	Cross	226	2.756863	0.221873	0.362731	0.010886
KAT2B	Cross	194	2.905322	0.171731	0.344196	0.012027
ICAM1	Cross	157	2.963585	0.148503	0.337429	0.013351
GANAB	Cross	94	3.021289	0.081593	0.330985	0.023191
UCHL3	Cross	90	3.022969	0.083068	0.330801	0.02
LGALS1	Cross	88	2.963025	0.082779	0.337493	0.024295
KRT1	Cross	82	2.893557	0.102386	0.345595	0.018179
FMNL1	Cross	69	2.961345	0.074724	0.337684	0.020059
IGHA1	Cross	68	3.259384	0.065726	0.306806	0.019608
CXCR4	Cross	65	3.032493	0.058645	0.329762	0.025835
PRDX4	Cross	63	3.029692	0.04942	0.330067	0.032967
TSPAN5	Cross	61	3.063866	0.06572	0.326385	0.018822
USP1	Cross	56	3.169188	0.049733	0.315538	0.027562
MMP9	Cross	48	3.67395	0.045968	0.272187	0.022287
IGHM	Cross	47	3.313725	0.036144	0.301775	0.026716
CORO1A	Cross	46	3.014566	0.043594	0.331723	0.024953
UBC	Cross	45	2.24986	0.439259	0.444472	0.027133
WIPF1	Cross	39	3.105882	0.033637	0.32197	0.038703
GPR183	Cross	36	3.210084	0.038177	0.311518	0.028409
RORA	Cross	35	3.156303	0.039584	0.316826	0.035945

## Data Availability

The datasets used and/or analyzed during the current study are available from the corresponding author on reasonable request.

## Abbreviations

AUC: Area under the curve
CP: Chronic periodontitis
DEG: Differentially Expressed Genes
GEO: Gene Expression Omnibus
PPI: Protein-protein interaction
ROC: Receiver Operating Characteristics
VTE: Venous thromboembolism.
